# The CogBIAS longitudinal study protocol: cognitive and genetic factors influencing psychological functioning in adolescence

**DOI:** 10.1186/s40359-017-0210-3

**Published:** 2017-12-29

**Authors:** Charlotte Booth, Annabel Songco, Sam Parsons, Lauren Heathcote, John Vincent, Robert Keers, Elaine Fox

**Affiliations:** 1 Department of Experimental Psychology, University of Oxford, New Richards Building, Oxford, Headington OX3 7LG UK; 2 Department of Anaesthesiology, Perioperative and Pain Medicine, Stanford University, Stanford, CA USA; 3 Department of Biological and Experimental Psychology, Queen Mary University London, London, UK

**Keywords:** Cognitive bias, Genetic variation, Polygenic sensitivity scores, Longitudinal, Adolescents, Psychopathology, Anxiety, Depression, Impulsivity

## Abstract

**Background:**

Optimal psychological development is dependent upon a complex interplay between individual and situational factors. Investigating the development of these factors in adolescence will help to improve understanding of emotional vulnerability and resilience. The CogBIAS longitudinal study (CogBIAS-L-S) aims to combine cognitive and genetic approaches to investigate risk and protective factors associated with the development of mood and impulsivity-related outcomes in an adolescent sample.

**Methods:**

CogBIAS-L-S is a three-wave longitudinal study of typically developing adolescents conducted over 4 years, with data collection at age 12, 14 and 16. At each wave participants will undergo multiple assessments including a range of selective cognitive processing tasks (e.g. attention bias, interpretation bias, memory bias) and psychological self-report measures (e.g. anxiety, depression, resilience). Saliva samples will also be collected at the baseline assessment for genetic analyses. Multilevel statistical analyses will be performed to investigate the developmental trajectory of cognitive biases on psychological functioning, as well as the influence of genetic moderation on these relationships.

**Discussion:**

CogBIAS-L-S represents the first longitudinal study to assess multiple cognitive biases across adolescent development and the largest study of its kind to collect genetic data. It therefore provides a unique opportunity to understand how genes and the environment influence the development and maintenance of cognitive biases and provide insight into risk and protective factors that may be key targets for intervention.

## Background

Genetic variation and individual differences in selective cognitive biases (CBs) have been associated with psychological functioning in largely independent lines of research. The aim of the CogBIAS longitudinal study (CogBIAS-L-S) is to encourage the integration of these two fields of research in order to investigate the *cognitive* and *genetic* factors that are involved in the development of emotional vulnerability and resilience in a healthy adolescent sample. The fundamental hypothesis in the field of cognitive bias research is that CBs are habitual, deeply engrained ways of responding to affective information, that are associated with emotional vulnerability and resilience. The fundamental hypothesis in the field of genetic psychiatry research is that genetic and environmental factors interact to increase risk for psychopathology. The CogBIAS hypothesis [1] combines and extends these hypotheses by stating that at least some gene–by-environment interaction (GxE) effects on psychological functioning may be mediated by individual differences in CBs, and that certain genetic profiles may represent heightened sensitivity to the learning environment in a “for better or for worse” manner [2, 3]. Some evidence indicates that allelic variation on genes that are active in the brain are likely to play a role in how easy or difficult it is to develop such “negative” or “enhancing” CBs [4], so the identification of genetic profiles and how they relate to the development of CBs is a vital first step in understanding pathways to psychopathology and wellbeing. This research, if successful, could inform the development of future personalized interventions designed to improve emotion regulation skills and boost a more resilient cognitive style [1, 5].

Adolescence is considered to be a risky developmental period, as prevalence and onset of depression and anxiety increases significantly during this time [6, 7]. Emotional problems developing during adolescence can have extremely deleterious effects on subsequent development and there is a high probability of disorder reoccurrence in adulthood [7]. Many significant neurodevelopmental changes take place during adolescence, which lead to dramatic social reorientation and result in changes in motivation, as well as heightened affective responding [8–11]. This research pinpoints adolescence as a period of heightened sensitivity, particularly to the social environment. More research is needed to elucidate neurocognitive mechanisms associated with emotional vulnerability and resilience during this age period [12, 13]. There is growing evidence that CBs contribute to the onset and prevalence of early psychopathology [13–15], however much of this research is correlational and often focuses on specific CBs (e.g., biased attention) in association with specific emotional disorders (e.g., anxiety). CogBIAS-L-S aims to provide a broader picture by assessing a multitude of CBs in a large sample of healthy adolescents at three narrow time points, in order to investigate the developmental trajectory of a range of CBs on psychopathological, as well as resilient outcomes during normal development.

In keeping with the CogBIAS hypothesis, as well as evidence from epidemiological research that highlights the importance of GxE on psychological functioning [16], we will also measure genetic variation and subjective ratings of life experiences, in order to test the hypothesis that heightened biological sensitivity predicts maladaptive outcomes, mediated by negative CBs in combination with adversity, as well as adaptive outcomes, mediated by enhancing or protective CBs in combination with supportive environments [1]. The integration of cognitive and genetic methods will provide important new insights into psychological functioning across adolescence.

### Cognitive biases

The evidence that emotional vulnerability is associated with CBs that magnify threat-related information and negativity, relative to benign or positive information, is strong [13, 17–21]. It has been proposed that these CBs become habitual and automatic and so, over time, result in deeply engrained ways of thinking that infuse the brain with a negative processing style. As most cognitive and neural processes operate automatically, and at an implicit level, such CBs are therefore extremely difficult to undo [1]. Thus, a *downward spiral* of negative CBs leads to a preponderance of negative over positive emotions that, in time, can result in more entrenched negative biases. This sequence is a hallmark of emotional vulnerability that, in susceptible people, can all too easily tip into a variety of emotional disorders such as anxiety and depression. A contrasting *upward spiral* of positivity is characteristic of emotional resilience, and, as enhancing CBs and processes focus selectively on the positive, rather than the negative and benign aspects of life, positive emotions tend to gradually dominate leading to an upwards spiral that can boost flourishing and optimal mental health. Those who are vulnerable and fragile, and those who enjoy optimal mental health, experience these downward or upward patterns on a regular basis, which unfold into deeply habitual cognitive and neural processes that are likely to have a profound influence on a person’s life trajectory.

Selective CBs in attention, interpretation and memory are often associated with mood-related outcomes [13, 17, 18, 20]. Attention bias refers to the automatic preferential processing of salient information in the environment. While attention biases towards threat-related information have been primarily associated with anxiety in both adults [18] and children [17], biased attention towards negative stimuli is also characteristic of depression [22]. Conversely, attention bias for positive stimuli may represent a protective bias related to resilience [23]. Interpretation bias refers to the tendency to interpret inherently ambiguous situations as either positive or negative, and has been associated with both anxiety and depression [24]. Memory bias refers to the tendency to selectively remember positive or negative information, and a memory bias, particularly for negative self-referent information, has been shown to be characteristic of depression [20]. While previous research has tended to assess mood-related CBs in isolation, CogBIAS-L-S aims to integrate the measurement of CBs in order to understand their relative importance on psychopathological, as well as resilient outcomes. Investigating the development of multiple CBs in this way will allow us to test the *combined cognitive bias hypothesis* [25], which posits that CBs do not occur in isolation, but rather influence one another and interact to maintain psychopathological outcomes. The current study will add to the literature by providing a rich data-set of multiple CBs at three narrow time-points across adolescent development.

In contrast to biases in attention, interpretation and memory, CBs in action-tendency to approach reward related stimuli have been associated with impulsivity-related outcomes [26, 27]. To illustrate, in an independent field of research, CBs in action-tendency to approach reward related stimuli have been implicated in the development of addictions, such as high levels of drinking behaviour [26, 28]. This research has recently been extended to the food and obesity literature, and it has been found that food activates the same neural reward substrates as addictive drugs [29]. Action-tendencies to approach food stimuli in combination with low cognitive control have been shown to predict overeating [27]. It is theorised that increased sensitivity to reward related cues coupled with low cognitive control predicts externalising problems, such as overeating and substance misuse [26]. Until recently almost all research on action-tendencies has been conducted in adult samples, therefore not only will this study extend the research to adolescent populations, it will offer the first integration of research between CBs in mood-related outcomes with CBs in action-tendencies towards reward, which should facilitate the integration of these two fields. CogBIAS-L-S will investigate how such CBs are related to mood-related self-report variables on the one hand, and impulsivity-related variables on the other, including self-reported maladaptive eating and risk-taking.

### Genetic variation

The role of genetics in psychological functioning has been investigated with a variety of methods, such as twin studies and molecular genetic studies. Twin studies have uncovered the relative contribution of genes to psychological disorders and traits. For example, across many studies of adult twin populations, it is estimated that about 40% of variance in anxiety and depression can be explained by genetic factors, with the remaining variance explained by non-shared environmental factors [30, 31]. This heritability estimate is similar in older adolescent populations, while the shared environment plays more of a role in childhood [16, 32]. Twin and family studies also show that genes and environments do not operate in isolation, but work together through several forms of complex interplay including gene-environment correlation (rGE) and gene-environment interaction (GxE). For example, those at a high genetic risk of depression have been shown to be more likely to experience psychosocial adversity (rGE) and be more sensitive to its effects (GxE) than those with a low genetic risk [33].

Molecular genetic evidence for GxE has been reported for several genetic variants across multiple candidate genes. One of the most extensively researched variants in relation to depression is the serotonin transporter linked polymorphic region (5-HTTLPR), a 43 bp insertion/deletion polymorphism in the 5′ promoter region of the serotonin transporter gene which results in a short (*S*) and long (*L*) allele. There is evidence that the variant is functional with the *S* allele leading to a 50% reduction in serotonin expression and consequently higher concentrations of serotonin in the synaptic cleft [34]. In a seminal study in 2003, Caspi et al. reported that individuals with the *S* allele of the 5-HTTLPR were at an increased risk of depression, following life stress or childhood maltreatment. In contrast, those with the *L* allele appeared to be protected from the negative effects of adversity [35]. These findings have been subsequently replicated and extended to several further phenotypes including anxiety sensitivity [36] and depressive symptoms in adolescence [37]. Similar findings have also been reported for other candidate genes implicated in the stress response system (FKBP5, NR3C1) [38, 39], dopamine transmission system (DRD2) [40] and neurogenesis factor (BDNF) [41], amongst others. Although some promising findings with regard to GxE have been reported [42], the mechanisms of GxE are poorly understood, which has led some researchers to question the robustness of such findings [43]. More research is needed to elucidate the specific genetic variants and environmental conditions that give rise to GxE effects.

The differential effects of stress by genetic factors identified in GxE studies were originally conceptualised in diathesis-stress models. This model states that certain genes predispose individuals to the negative effects of adversity leading to mental illness [44]. However, an extended version of this theory is the “differential susceptibly hypothesis” (DSH), which posits that rather than risk factors alone, genetic variants may increase susceptibility to both negative and positive environments “for better *and* for worse” [2, 3]. This suggests that while the most biologically sensitive individuals will show adverse outcomes in combination with adverse environments, they will also benefit disproportionately from supportive and enriching environments [2, 3].

Many genetic variants implicated in GxE show patterns of association consistent with the DSH, with particularly promising findings from intervention studies [45]. Nevertheless, findings have failed to replicate consistently, leading to both positive [46] and negative meta-analyses [47]. One explanation for these findings is that sensitivity to the environment, like other psychological phenotypes, is a polygenic trait and results from the additive effects of multiple genetic variants of small effect [48]. Polygenic Sensitivity Scores (PSS) provide a marker of biological sensitivity to the environment that can be derived from cumulating alleles associated with heightened sensitivity into one score. For example, Belsky and Beaver [49] created a PSS based on five candidate genes affecting the serotonin and dopamine systems, and found that individuals with the highest number of sensitivity alleles showed both the best and worst psychological outcomes relative to childhood experiences, supporting the DSH. This has also been shown in relation to resilience in childhood, as a PSS derived from multiple genes affecting neurotransmission predicted the best and worst psychological functioning relative to the presence or absence of childhood maltreatment [50]. The availability of genome-wide data has allowed for more recent studies to extend this approach beyond candidate genes to derive PSSs from genetic variants across the entire genome. For example, a recent study using a novel approach comparing identical twins derived a genome-wide PSS that significantly moderated the effects of parenting on emotional problems in a manner consistent with the DSH and was a good predictor of response to psychological treatments in children with anxiety disorders [51].

In CogBIAS-L-S, we will investigate the interaction between genetic variants and positive and negative experiences across adolescent development. This will allow us to assess whether biological sensitivity predicts “for better and for worse” outcomes in this age group and to identify new genetic factors associated with psychological and cognitive phenotypes that may remain hidden by interactive effects with the environment [52]. The availability of whole-genome genetic data will allow us to investigate the role of current and emerging genome-wide PSSs as well as candidate genetic variants previously shown to have a significant effect on mood and impulsivity related outcomes.

### The CogBIAS hypothesis

**Fig. 1:**
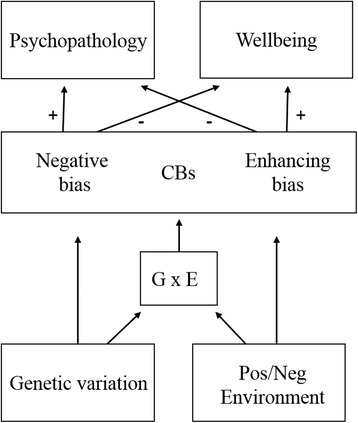
Simplified version of the CogBIAS hypothesis [1] showing the effect of gene-by-environment interaction (GxE) on psychological functioning mediated by negative and enhancing cognitive biases (CBs)

The CogBIAS hypothesis [1] offers a theoretical model of psychological functioning, which integrates cognitive and genetic research. As depicted in Fig. 1, CBs act as mediating mechanisms in the pathway to psychological functioning between genetic moderation of the environment (GxE). The development of negative or enhancing CBs is dependent on biological sensitivity to the effects of the environment, which can be either supportive or unsupportive, leading to different outcomes. While negative CBs increase risk for psychopathology and decrease wellbeing, enhancing CBs are likely to increase wellbeing and decrease risk for psychopathology. CogBIAS-L-S will be able to test this hypothesis, as an in-depth assessment of CBs and psychological variables will be taken at three narrow time points across adolescence, as well as genome-wide testing conducted at the beginning of the study. A similar recent model of adolescent psychopathology has highlighted the importance of CBs as mediating factors between genetic risk and anxiety/depression outcomes [13]. However, these hypotheses have yet to be tested using a multitude of CBs and respective outcomes related to both psychological vulnerability and resilience.

Preliminary evidence supports the potential role of genetics in the development of CBs. In an earlier study, strong attention biases towards threat-related images or towards positive images was trained in 5HTTLPR *S* allele carriers, whilst no training effects were observed in those carrying the *L* version of the gene, suggesting that the *S* allele may act as a sensitivity gene that moderates the learning environment in a “for better and for worse” manner [4]. In support of this, a recent meta-analysis has found an association between attention bias for threat and the *S* allele with a medium effect size [53]. Attention bias should be considered a dynamic response to the environment, as vigilance for threat can be adaptive under stressful conditions [54], therefore investigating attention bias in relation to GxE is an important next step. Furthermore, the general consensus in the literature is that moving away from single candidate gene studies in favour of assessing polygenic effects on outcomes is appropriate, as comprehensive aggregated scores, such as PSS, are likely to explain more variance in behaviour than single genes [55]. Recent developments in PSS will allow us to investigate more complex models of GxE influencing CBs in attention, interpretation, memory and action-tendency, and how this relates to adolescent psychological functioning.

## Method

### Study design and aims

CogBIAS-L-S will follow a large sample of over 500 adolescents for approximately 4 years and will test participants at three time points when they are 12, 14 and 16, in order to assess CBs (in attention, interpretation, memory and action-tendency), life experiences, and a range of subjective measures (including anxiety, depression, resilience, impulsivity and risk-taking) at each time point. Genetic variation will be assessed once at Wave 1. This research design is focused on a narrow developmental period and includes a wide range of cognitive and subjective factors, which will help to identify psychological profiles associated with emotional vulnerability and resilience, and insight into risk and protective factors that may be key targets for intervention strategies designed to improve psychological functioning.

#### Sample size

A sample size of 500 is considered highly powered for detecting even small effects in the cognitive bias domain. Genetic studies require larger samples, due to small effect sizes and expected complex interactions with multiple genes and the environment. Previous GxE studies have been criticised for using small sample sizes (e.g. *N* < 200), which may be statistically unstable, therefore a minimum sample size of 300 has been suggested to be adequate for such studies [47]. We aimed for a sample size of 500 to balance the need for a large enough sample to detect GxE with the feasibility to collect detailed psychological data. We also aimed for a large sample to allow for the potential decrease in sample size with each wave of assessment.

### Recruitment

Participants will be recruited through their schools, by writing emails to head teachers or psychology teachers describing the aims of the study, the commitment needed from the school, and offering to work closely with the school on extracurricular projects, such as giving talks to pupils and organising work experience opportunities in our research lab. We aim to recruit ten cohorts from a variety of schools in the South England area, including private and comprehensive schools, and equal numbers of boys and girls.

#### Inclusion criteria

Inclusion criteria for the study encompasses having a parent and adolescent able to give written informed consent/assent, being aged between 12 and 16, being able to speak English fluently, as well as attending a secondary school in England that is taking part in the study.

#### Exclusion criteria

Exclusion criteria includes currently suffering with a psychological disorder or any neurological impairment or learning disability that would make them unable to take part. These criteria will be indicated by parent self-report.

### Procedure

Testing will take place at participant’s schools, or in some cases at the Department of Experimental Psychology, University of Oxford. Each assessment wave comprises two sessions lasting 1 h each, which will either be completed back-to-back, or on different days, depending on the availability to book testing space. Participants will complete test sessions in small groups in computer labs and will be asked to conduct assessments in exam conditions, therefore being silent and not looking at their neighbour’s computer screen. Participants will be asked to give written assent after the study procedure is explained to them. They will complete a batch of cognitive tasks, followed by questionnaires in each session in the same order. Table 1 outlines the testing procedure undertaken at Wave 1. Saliva samples will be collected at the end of test session two, only once at Wave 1.

**Table 1:** Testing procedure for CogBIAS longitudinal study (Wave 1)

Session one	Session two
Cognitive tasks:	Cognitive tasks:
Flanker task	Memory bias task
Dot-probe task	Balloon Analogue Risk task
Adolescent Interpretation and Belief questionnaire	Approach bias for food
Questionnaires:	Questionnaires:
Self-esteem	Impulsivity
Worry	BIS / BAS
Anxiety & Depression	Risk taking
Peer victimisation	Eating behavior
Experiences	Mental Health
Rumination	Resilience
Pain questionnaires	+
	Saliva sample

### Measures

#### Questionnaires

##### Anxiety and depression

The Revised Children’s Anxiety and Depression Scale - Short Form (RCADS-SF) [56] is a 25-item self-report questionnaire used to assess anxiety and depression symptoms. The RCADS-SF comprises 6 subscales corresponding to separation anxiety, generalized anxiety, panic disorder, social anxiety, obsessive compulsive disorder, and depression. Items are scored on a 4-point Likert scale ranging from 0 (“Never”) to 3 (“Always”). The items corresponding to anxiety are summed to yield an *Anxiety Total Score*, as well as summing each item corresponding to each anxiety subscale (i.e., separation anxiety, generalized anxiety, panic disorder, social anxiety, and obsessive-compulsive disorder), and the items related to Depression are summed to calculate a *Depression Total Score*. Higher scores indicate higher symptoms of anxiety and depression in adolescents. The RCADS-SF is derived from the original 47-item questionnaire [57], and has shown to have good reliability and validity in children and adolescents [58].

##### Worry

The Penn State Worry Questionnaire for Children (PSWQ-C) [59] is a 14-item self-report measure used to assess the tendency to worry in children aged 6 to 18 years old. Examples of items include “My worries really bother me” and “I know I shouldn’t worry, but I just can’t help it.” Each item is rated on a 4-point Likert scale from 0 (“Never true”) to 3 (“Always true”) and a *Worry Total Score* is calculated by summing the items. Higher scores on the PSWQ-C indicate more frequent and uncontrollable worries. In adolescent samples, the PSWQ-C has excellent internal consistency, good convergent and discriminant validity, and test-retest reliability in clinical and non-clinical samples [59–61].

##### Rumination

The Children’s Response Style Scale (CRSS) [62] assesses rumination and coping styles when confronted with low mood. The 20-item self-report questionnaire is comprised of two subscales to reflect *Rumination* (e.g. “When I feel sad, I think back to other times I have felt this way”) and *Distraction* (e.g. “When I feel sad, I think about something I did a little while ago that was a lot of fun”). Participants rate each item on a 10-point Likert scale ranging from 0 (“Never”) to 10 (“Always”). A *Rumination Total Score* and a *Distraction Total Score* are computed by summing across relevant items. Previous research has demonstrated good internal consistency for the two subscales, good test-retest reliability, and good validity with meaningful associations with depression and other response style measures [62].

##### Self-esteem

The Rosenberg Self-esteem Scale (RSE) [63] assesses levels of self-esteem. Participants are asked to rate 10 statements relating to self-worth and self-acceptance (e.g. “I feel that I have a number of good qualities”) on a 4-point Likert scale ranging from 0 “Strongly disagree” to 3 “Strongly agree”. The items are summed to create a *Self-esteem Total Score*, with higher scores reflecting greater levels of self-esteem. Previous research has shown good internal reliability [64, 65] and validity in adolescent populations [66].

##### Mental health

The Mental Health Continuum - Short Form (MHC-SF) [67] contains 14-items to assess wellbeing. The MHC-SF has three subscales that include *Emotional, Psychological,* and *Social Wellbeing*, in order to create a composite measure of *Total Wellbeing*. Participants rate how often they have experienced each of the items in the past month, on a 6-point Likert scale from 0 (“Never”) to 5 (“Every day”). Sum scores are created for each subscale, as well as a *Total Wellbeing* score, with higher scores reflecting greater wellbeing. The MHC-SF has shown high internal consistency and discriminant validity [68, 69].

##### Resilience

The Connor-Davidson Resilience Scale – Short form (CD-RISC-SF) [70] is a 10-item scale designed to measure trait resilience (e.g. “I believe I can achieve my goals even if there are obstacles”). Participants are asked to rate how each item applies to them in the past month on a 5-point scale from 0 (“Not true at all”) to 4 (“True nearly all the time”). The items are summed to create a *Resilience Total Score*, with higher scores indicating higher levels of resilience. The scale has demonstrated strong psychometric properties with good internal validity, reliability and validity [70].

##### Peer victimisation

The Multidimensional Peer Victimization Scale (MPVS) [71] assesses bullying. The 16-item scale consists of four subscales that relate to different forms of bullying. The subscales include *Physical Victimization* (e.g. “Beat me up”), *Verbal Victimization* (e.g. “Swore at me”), *Social manipulation* (e.g. “Tried to make my friends turn against me”), and *Property Vandalism* (e.g. “Deliberately damaged some property of mine”). Participants are asked to rate how often each item has happened to them in the past 12 months by a fellow classmate on a 3-point Likert scale including 0 (“Not at all”), 1 (“Once”) and 2 (“More than once”). A *Victimization Total Score* is calculated by summing together the 16 items. In addition, scores for each subscale (*Physical, Verbal, Social,* and *Vandalism*) are calculated by summing the corresponding items. The MPVS has demonstrated good internal reliability for each of its four subscales and has shown to be correlated with PTSD in children [71–73].

##### Life experiences

The Child Adolescent Survey of Experiences – Child version (CASE –C) [74] is a measure of negative and positive life events. The self-report questionnaire consists of 38 life events covering a broad range of stressful (an example item is “My parents split up”) and enjoyable (“I went on a special holiday”) experiences. Participants indicate whether each life event has occurred in the previous 12 months. Then each reported life event is rated on a 6-point Likert scale (1 = really bad, 2 = quite bad, 3 = a little bad, 4 = a little good, 5 = quite good, 6 = really good). Firstly, the number of *Positive Life Events* and *Negative Life Events* are calculated by summing the relevant life experiences, based on the respondent’s evaluation of whether it was a good or bad experience. Secondly, the impact of the life event is calculated by assigning 3 points for the responses “really good” and “really bad”, 2 points for the responses “quite bad” and “quite good”, and 1 point for the responses “a little bad” and “a little good”. The points are then summed to create a *Negative Impact Score* and a *Positive impact Score*. Higher scores indicate a greater positive or negative impact of the life event. Additionally, participants can report up to two extra significant life events that happened to them in the past 12 months, which are incorporated into the total score. The CASE-C has good psychometric properties, with previous studies indicating that it can discriminate anxious children from healthy controls [75] and detect significant associations between negative life events and depression in adolescent girls [76].

##### Pain experiences

The Oxford Adolescent Pain Questionnaire (OAPQ) is a novel, 17-item self-report measure that assesses young people’s recent and chronic pain experiences. The OAPQ is a collection of individual items largely taken and adapted from existing questionnaires that assess a variety of pain experiences. In particular, a number of items were adapted from the Brief Pain Inventory [77] (e.g., 11-point visual analogue scales indicating average and worst pain intensity, and pain frequency, in the preceding months and weeks). Participants were also asked questions about the impact of any ‘aches or pains’ they had recently experienced (e.g. “How much have you missed out on activities that other people your age do, because of pain, in the last 4 weeks”). Additional items were also included to assess participants’ pain body locations and pain beliefs.

##### Pain Catastrophizing

The Pain Catastrophizing Scale – Child Version (PCS-C) is a 13-item self-report measure assessing young people’s magnification of pain, rumination about pain, and feelings of helplessness when in pain (e.g. “When I am in pain, I become afraid that the pain will get worse”) [78]. Higher scores on the PCS-C indicate more catastrophic thoughts about pain. The PCS-C has good reliability and validity for children older than 9 years [78].

##### Impulsive behaviour

The UPPS-R-Child version (UPPS-R-C) [79] will be used to assess impulsivity. The 32-item questionnaire comprises four factors of impulsivity including *Lack of Premeditation*, *Lack of Perseverance*, *Sensation seeking*, and *Negative Urgency*. Lack of premeditation refers to a difficulty in controlling impulses (e.g. “I tend to blurt things out without thinking”). Lack of perseverance refers to a difficulty in completing tasks (e.g. “I tend to get things done on time”- reverse scored). Sensation-seeking refers to the preference for doing exciting and thrilling activities (e.g. “I would enjoy water skiing”). Negative urgency refers to the tendency to act impulsively in response to negative emotional states (e.g. “When I feel bad, I often do things I later regret in order to feel better now”). Participants are asked to rate each item based on how best the statement describes them on a 4-point Likert scale ranging from 1 (“Not at all like me”) to 4 (“Very much like me”). Total scores for the subscales are calculated by summing the relevant items. The measure has good psychometric properties and has demonstrated good internal consistency and reliability [79].

##### Behavioural inhibition and behavioural activation

The BIS/BAS Scale Child Version [80] will be used to measure the propensity to approach reward (behavioural activation) and also to avoid things that are unpleasant or aversive (behavioural inhibition). The 20-item self-report questionnaire consists of four subscales; a *Behavioural Inhibition scale* and three behavioural activation scales, which include *Reward Responsiveness, Drive,* and *Fun Seeking*. Each item is rated on a 4-point Likert scale ranging from 0 (“Not true”) to 3 (“Very true”). The measure has previously shown good psychometric properties [81, 82].

##### Risk taking

The Risk Involvement and Perception Scale [83] will be used to assess adolescent risk-taking behaviour. The current study used an adapted version with 14 of the original 23 items, thought to be appropriate for the current young adolescent sample. The measure consists of three subscales, *Involvement* in risk behaviour, *Risk Perception* of the negative consequences, and *Benefit Perception* of the benefits associated with the risk behaviour. The 14 items included risk behaviours that are illegal for all people, those that are inappropriate for young people, and those that involve some measure of social and physical risk (e.g. drinking alcohol, skipping school). Participants rate how frequently they have undertaken the risk behaviour, as well as rating the ‘consequences’ and ‘benefits’ of each behaviour on an 8-point Likert scale ranging from 0 (“Not bad at all/Not good at all”) to 8 (“Really good/Really bad”). The *Involvement* subscale is calculated by averaging the frequency of risk behaviours. The *Risk Perception* and *Benefit Perception* subscales are calculated by averaging the corresponding negative and positive ratings. The measure demonstrates good internal validity for each subscale as well as good test-retest reliability and validity [83].

##### Eating behaviour

The Three Factor Eating Questionnaire (TFEQ – R18) [84] will be used to measure cognitive and behavioural components of eating. The 18-item questionnaire consists of three subscales, *Cognitive Restraint, Uncontrolled Eating* and *Emotional Eating*. Cognitive restraint refers to the conscious restriction of food intake in order to control body weight. Uncontrolled eating is the tendency to eat more than usual due to a loss of control. Emotional eating refers to the inability to control eating in response to emotional cues. Participants rate items on a 4-point scale (0 = “Definitely false”, 1 = “Mostly false”, 2 = “Mostly true”, 3 = “Definitely true”). Individual subscale scores are calculated by summing the corresponding items, with higher scores reflecting greater cognitive restraint, uncontrolled, or emotional eating. The TFEQ-R18 has good psychometric properties and has been shown to predict unhealthy eating and obesity [84–86].

#### Cognitive tasks

##### Attention bias

A pictorial *dot-probe task* [87] with faces will assess attention bias to three separate emotional categories. Threat bias will be assessed with angry faces, positivity bias with happy faces, and pain/empathy bias with pain faces. The task comprises three blocks corresponding to each of these categories. Within each emotion block 56 trials will present an emotional face paired with a matched neutral face (same actor) for 500 ms, followed by a probe for 3000 ms either behind the emotional face (congruent trials) or behind the neutral face (incongruent trials), therefore attention bias for emotion can be inferred if RT is faster on congruent compared to incongruent trials. The faces were chosen from the STOIC faces database [88] which is a validated set of 10 actors expressing six basic emotions. We chose seven actors (four male: three female) and four emotions (neutral, anger, happiness, pain) to make up our task of 168 trials – each actor was shown 8 times in each block. The faces are presented in greyscale with no hair or jawline showing on a grey background. Pictures are 230 × 230 pixels in size and presented approximately 10 degrees visual angle apart. Probes are the letters ‘Z’ and ‘M’ corresponding to the correct response, which are presented equally on the left or right, to increase task difficulty and encourage attentional engagement. The inter-trial interval (ITI) is 500 ms, followed by a fixation cross presented for 500 ms to signal the start of a new trial. Participants are instructed to focus on the fixation cross and ignore the faces, but respond to the probe as fast as they can, without compromising their accuracy. An error message is shown if participants make an incorrect response or if no response is made within 3000 ms. Block order is counterbalanced across participants and a rest period of 30,000 ms with a timer is displayed between blocks. Participants also complete a practice block with 8 trials depicting only the probe and 16 trials with neutral-neutral face pairings, which are not analysed. Bias indices are calculated separately for threat, positivity and empathy, as the difference in RT between congruent and incongruent trials (high numbers reflecting attentional orienting). Incorrect trials and trials that are responded to <200 ms or >3000 ms or 3 SDs from each participants mean RT for each trial type and emotion category will not be analysed. Participants who exceed an error rate near chance will be excluded. Visual analogue scales (VAS) will be presented immediately before and after the task to assess mood. Participants will be asked to rate how happy they feel and how sad they feel using a 10-point sliding scale.

##### Memory bias

*The Self-Referential Encoding Task* (SRET) [89] will be used to assess memory bias for self-referential words. The task comprises three phases – an encoding phase, a distraction phase, and an incidental free recall phase. In the encoding phase, self-referent adjectives are displayed on the screen for 200 ms, followed by the caption “Describes me?” which is presented below the word at which point participants can respond with either yes or no using the “Y” and “N” keys. A new word is presented after a valid response is made. The word list comprises 22 positive (e.g. “cheerful”, “attractive”, “funny”) and 22 negative (e.g. “scared”, “unhappy”, “boring”) self-referent adjectives that have previously been validated for use in an adolescent sample and were matched on word length and recognisability [89]. In the distraction phase, participants are instructed to solve three simple mathematics equations by typing their response into a short answer box. Responses do not have to be correct and will not be analysed. In the incidental free recall phase, participants are instructed to type as many words as they can remember from the “Describes me” task, regardless of whether they endorsed the word, into a long answer box. They are given 3 min for recall, at which point the task ends. We will calculate *positive memory bias* as the total number of positive words that are endorsed and recalled, and *negative memory bias* as the total number of negative words that are endorsed and recalled [90]. Negative memory bias has previously been computed as one score, dividing the number of endorsed and recalled negative words by the total number of endorsed and recalled words [91], however this method is better suited to research in clinical populations, as many participants in our sample will not be expected to endorse many negative words and we are interested in assessing variability in positivity bias, which could explain resilient functioning.

##### Interpretation bias

*The Adolescent Interpretation and Belief Questionnaire* (AIBQ) [92] will be used to assess interpretation bias to hypothetical positive and negative social and non-social situations. Participants read 10 ambiguous scenarios and are asked to imagine that the situations are happening to them. They are then shown three thoughts that could arise in response to the situation and are asked to rate how much each thought would be likely to pop into their head using a 5-point Likert scale (1 = does not pop in my mind, 3 = might pop in my mind, 5 = definitely pops in my mind), they are then asked which would be the most likely thought to pop into their mind using a forced-choice procedure. The interpretations for each scenario are either neutral, positive or negative. Interpretations for each situation are presented in a fixed random order. Outcome variables are created for *Positive Interpretation (Social)* as the summation of the ratings on the positive social interpretations divided by the 5 social situations, for *Negative Interpretation (Social)* as the summation of the ratings on the negative social interpretations divided by the 5 social situations, for *Positive Interpretation (Non-Social)* as the summation of the ratings on the positive non-social interpretations divided by the 5 non-social situations, and for *Negative Interpretation (Non-Social)* as the summation of the ratings on the negative non-social interpretations divided by the 5 non-social situations. Outcome variables can also be calculated from the forced-choice questions by scoring positive choices as 1, neutral choices as 2, and negative choices as 3, and then creating a score for *Social Interpretation Bias* by summing the choices from the social items, and for *Non-social Interpretation Bias* by summing the choices from the non-social items. For the current study we will focus on the four outcomes calculated using the Likert-scale question in order to increase variance, as well as to adhere to most previous research with the AIBQ [92, 93]. Scores can range from 1 (no bias) to 5 (strong bias) for each of the four outcome variables.

##### Risk-taking

*The Balloon Analogue and Risk Task* (BART) [94] will be used to assess risk-taking propensity. Participants are instructed to pump a computer-generated red balloon with a button press and gain 1 point for each pump, which they have to ‘bank’ in a points meter with a different button press before they feel the balloon will burst. Responses are made with a left mouse click on the respective button displayed on the screen – either below the balloon (which increases in size with each pump) or below the points meter (which increases in points). If balloons burst then no points can be won on that trial. Participants are instructed to get as many points as possible whilst being careful not to burst the balloons. They will complete 20 balloon trials, which have an average bursting point of 60 pumps (same for the first and second half of the task) and a range from 10 to 111. Pumping that exceeds the bursting point causes the balloon to explode into pieces across the screen. The balloon trial number is displayed at the bottom of the screen. The average number of pumps on balloons that did not burst will be used as an index of risk taking. This adjusted value is optimal to using the average number of pumps across all trials, because the outcome is not constrained by the bursting point, i.e. most participants would be expected to burst balloons with a very low bursting point [94].

##### Approach bias for food

We will use a *Stimulus-Response Compatibility* task (SRC) [95] task to assess automatic approach bias for food. Participants are instructed to approach or avoid different stimulus categories with a manikin using the up/down arrow keys. The task consists of two blocks – a food approach/non-food avoid block and a food avoid/non-food approach block – which are counterbalanced in order of presentation. A trial begins with a fixation cross in the centre of the screen (1000 ms), replaced by a stimulus (food or non-food picture) in the centre of the screen with a manikin (15 mm high) positioned 40 mm above or below the picture. There is a brief ITI (500 ms). Participants are instructed to either approach or avoid each stimulus type at the beginning of the task (i.e. stimulus type is task-relevant), and are instructed again after the end of the first block that the instructions have reversed. The task consists of 112 experimental trials in total. Approach and avoidance responses are made by pressing the up or down arrow keys. Responding causes the manikin to become animated and move in the direction of the arrow press. Each trial is completed when the participant has made three responses and the manikin either reaches the picture (approach trials) or reaches the top/bottom of the screen (avoid trials). Only the initial RT will be used for data analysis. Pictures were chosen from the food-pics database [96] which contains over 800 images of food and non-food items rated on perceptual characteristics and affective ratings. We chose 8 sweet snack food pictures (e.g. donut, ice-cream, grapes and blueberries) and 8 non-food miscellaneous household pictures (e.g. cushion, key, book and umbrella) that we matched for complexity, familiarity and valence. Approach bias for food will be calculated as the difference in RT in the approach food block and the avoid food block (high numbers reflecting a strong approach bias). Incorrect trials and trials that are responded to <200 ms or >3000 ms or 3 SDs from each participants mean RT for each trial type will not be analysed. Participants who exceed an error rate near chance will be excluded. VAS hunger scales will be presented immediately before and after the SRC task [97], to control for baseline hunger in later analyses.

##### Attention control

The Flanker task [98] will be used to assess an aspect of attention control known as response inhibition. Participants are instructed to respond to the direction of a target fish in the middle of the screen, whilst ignoring two fish on either side of the target fish. There are 116 experimental trials which are randomly and equally likely to be congruent trials – when the flanker fish points in the same direction as the target – or incongruent trials – when the flanker fish points in the opposite direction, causing interference. There are four trial types – target (left) congruent, target (right) congruent, target (left) incongruent, and target (right) incongruent. The stimuli are yellow fish with a faint black arrow embedded in the image (150 × 230 pixels) and are presented approximately 1 degree of visual angle apart on a white background. Participants will complete ten practice trials with only the target fish and ten practice trials with the flanker fish, which will not be analysed. Responses are made using the left/right strict inequality symbols (“<” and “>”) and participants are instructed to keep their index fingers on the response keys throughout the task so that they can respond as fast as possible. Participants are also instructed not to make any mistakes and an error message is displayed when an incorrect response is made (“Wrong response”) or when responses are slower than 2500 ms (“Too slow”). Error feedback is displayed for 1000 ms and the ITI is 1250 ms. A short rest period is given halfway through the task and a countdown clock is shown for 30,000 ms. Difference in RT between congruent and incongruent trials reflects response inhibition (high numbers reflecting strong interference). Error trials and trials <200 ms or >2500 ms will not be analysed, as well as trials that are 3 SDs from each participant mean RT for each trial type.

#### Body-mass-index

Body-mass-index (BMI) will be calculated (BMI: kg/m^2^) from measuring participant’s height (meters) and weight (kilograms) using a *Seca* portable height measure and *Salter* portable weight scales.

#### Genotyping

Saliva samples will be collected using *DNA Genotek Oragene OG-500* collection kits in accordance with the supplied instructions and genomic DNA extracted using an established protocol and stored at −80 °C. The samples will be genome-wide genotyped using the Illumina Human Omni express-24, which captures 710,000 single nucleotide polymorphisms (SNPs) from across the genome. This chip assays the majority of genetic variants implicated in sensitivity to the environment, either directly or through imputation. It is therefore considerably more cost effective and requires less DNA per variant than candidate-gene genotyping. Our genome-wide approach will also allow analyses using existing and emerging whole-genome polygenic scores and hypothesis-free genome-wide analyses on integration with further data. Genome-wide data will be subject to rigorous quality control using an established pipeline and additional SNPs imputed using the 1000 Genomes reference panel. In addition to genome-wide genotyping, we will also genotype several genetic variants implicated in sensitivity to the environment, which are not captured by genome-wide arrays including STin2 and DRD4 using established protocols. The 5-HTTLPR will be genotyped simultaneously with rs25531, a SNP proposed to modify the effects of the L allele on gene expression, using a two-stage method. In the first stage short and long alleles will be determined by polymerase chain reaction (PCR). In the second stage, the PCR product is incubated with a restriction enzyme (Msp1), which cuts the resulting product depending on the rs25531 genotype. Fragment lengths will be compared using gel electrophoresis and the specific combination of fragments produced will be used to determining genotype.

#### Statistical analysis

We will use a multilevel moderated mediation model [99] in order to test the CogBIAS hypothesis. This model will test whether CBs mediate the effect of the environment on the development of psychopathological outcomes across three time points and whether PSS moderates the influence of the environment on the development of CBs. For example, we will test whether negative experiences predict negative selective CBs, which in turn predict prevalence of depressive symptoms and whether a PSS based on depression-related genetic alleles moderates this relationship. Data from three time points will be used in the model, in order to assess these moderated mediated effects across adolescent development. A benefit of using a longitudinal design is the ability to test whether experiences and CBs in early adolescence predict future psychopathological outcomes, rather than only using a correlational design, which does not allow for interpretation of causality. As well as testing the CogBIAS hypothesis, which integrates cognitive and genetic research, we will also test more specific research questions within each of these topics, such as testing the combined cognitive bias hypothesis [25], which will largely look at correlations between the various CBs.

## Discussion

Investigating cognitive and genetic factors associated with psychological functioning across adolescent development will provide an important proof of principle for the integration of these two literatures. Recent reviews have highlighted that CBs have yet to be well integrated into the literature on biological factors predicting psychopathology [1, 13]. CogBIAS-L-S will allow the investigation of many research questions facilitating the integration of these two fields, as well as addressing many more specific research questions. The cognitive bias literature will benefit from the measurement of multiple CBs across three time points, which will allow for investigation of the combined cognitive bias hypothesis [25] and improve understanding of the developmental trajectory of anxiety, depression and emotional wellbeing and resilience during this important developmental period [13]. Further, developing a greater understanding of CBs and related disorders will lay the groundwork for the development of better intervention practices designed to improve psychological functioning. The genetic psychiatry literature will benefit from the ability to test novel PSS in interaction with environmental experiences across adolescence, which will improve understanding of GxE effects and heightened biological sensitivity to the environment across the lifespan. CogBIAS-L-S is the largest study of its kind to collect longitudinal data on multiple CBs, as well as comparative genetic data, which will provide important insight into the complex interplay between individual and situational factors predicting optimal development in adolescence.

### Study status

CogBIAS-L-S is currently on-going. Data collection for Wave 1 commenced in October 2014. Data has been collected so far up to Wave 2. Data collection for Wave 3 will cease by October 2018. Test sessions were spaced approximately 18 months apart.

### Recruitment

We contacted 51 schools in the South England area. Thirty percent of schools contacted were initially interested. The final sample included 20% of the schools contacted, which was made up of ten cohorts (from nine different schools). Once the schools had given permission for their pupils to be contacted to take part in the study, teachers arranged for information packs (containing a detailed parent information sheet, adolescent information sheet and parent consent form) to be sent home either electronically or in paper form. We received parental consent for 530 adolescents to take part. Test sessions were organised by each individual school cohort. The final sample tested at Wave 1 included 504 adolescents (*M* age = 12.9, *SD* = .9), of which 55.2% were female. The majority of cohorts were in year 8 (6:10), with the addition of two in year 7 and two in year 9. The majority of cohorts were from same-sex schools (6:10). Sample demographics (at Wave 1) for each cohort are presented in Table 2.

**Table 2:** Sample demographics for CogBIAS longitudinal study (Wave 1) by cohort group

Variable	Total	X1	X2	X3	X4	X5	X6	X7	X8	X9	X10
N	504	15	30	62	47	13	34	119	104	54	26
Age (*M, SD*)	12.9 (.9)	12.0 (.5)	11.3 (.4)	12.9 (.4)	12.9 (.3)	11.9 (.5)	12.3 (.5)	13.5 (.6)	12.6 (.7)	13.9 (.5)	12.7 (.5)
Year group (range)	7–9	7	7	8	8	8	8	9	8	9	8
Gender (% female)	55.2	40.0	50.0	100.0	100.0	100.0	47.1	0.0	100.0	0.0	57.7
Ethnicity (% Caucasian)	78.9	93.3	86.7	72.6	80.8	69.5	61.1	89.1	71.1	83.4	96.1
Mother’s education level (*M, SD*)	3.6 (1.3)	3.9 (1.4)	2.9 (1.4)	3.6 (1.1)	2.9 (1.4)	4.1 (1.4)	2.4 (1.1)	4.2 (1.1)	3.9 (1.0)	3.5 (1.5)	3.0 (1.2)

### Genotyping

Of the 504 participants assessed in Wave 1, 499 (99%) provided a saliva sample. Samples with an adequate yield of DNA following extraction (200 ng, *n* = 496) were run on the genome-wide array in accordance with the manufacturer’s instructions. Following rigorous quality control 594,667 SNPs and 491 individuals remained for analyses with a further 5,129,755 SNPs successfully imputed from the 1000 Genomes reference panel. A total of 496 participants (99.4% of those providing a saliva sample) were also successfully genotyped for the 5-HTTLPR and rs25531 using the two-stage method.

## Abbreviations

CBs: Cognitive biases
CogBIAS-L-S: CogBIAS longitudinal study
DSH: Differential susceptibility hypothesis
GrE: Gene-by-environment correlation
GxE: Gene-by-environment interaction
PSS: Polygenic sensitivity scores
